# GroEL—A Versatile Chaperone for Engineering and a Plethora of Applications

**DOI:** 10.3390/biom12050607

**Published:** 2022-04-19

**Authors:** Maria S. Yurkova, Alexey N. Fedorov

**Affiliations:** Bach Institute of Biochemistry, Research Center of Biotechnology of the Russian Academy of Sciences. 33, Bld. 2 Leninsky Ave., Moscow 119071, Russia; info@fbras.ru

**Keywords:** chaperonin, GroEL, engineering of chaperones, chaperones in biotechnology

## Abstract

Chaperones play a vital role in the life of cells by facilitating the correct folding of other proteins and maintaining them in a functional state, being themselves, as a rule, more stable than the rest of cell proteins. Their functional properties naturally tempt investigators to actively adapt them for biotechnology needs. This review will mostly focus on the applications found for the bacterial chaperonin GroE and its counterparts from other organisms, in biotechnology or for research purposes, both in their engineered or intact versions.

## 1. Introduction

Chaperones’ main function is assisting in protein folding, both co-transalational or in any stress conditions [1], and chaperonins are part of the chaperone’s superfamily. The chaperonin family is usually divided into two subfamilies: group I (bacterial chaperonin GroEL and co-chaperonin GroES; mitochondrial HSP60 and co-chaperonin Hsp10; chloroplast Cpn60 and co-chaperonin Cpn10/20) and group II (eukaryotic chaperonin-containing TCP-1, CCT; archaeal thermosome) [2]. The bacterial system of chaperonin GroEL in the complex with co-chaperonin GroES (GroE complex) is by far the most studied [3,4,5,6]. In most organisms, GroE exists in the form of an oligomer consisting of two rings formed by seven monomers each and stabilized by the ring of seven monomers of co-chaperonin GroES. GroEL may be engineered for stabilization, biosynthesis, or soluble expression of polypeptides in any of its forms: as a full-sized oligomer, a monomer, or a separate apical domain, called a minichaperone. All these cases are described in detail below.

**Figure 1:**
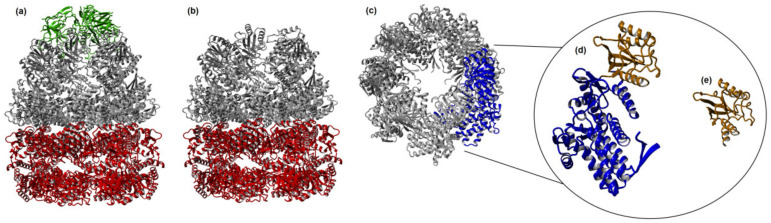
The forms of chaperonin GroEL that find applications in biotechnology: (**a**) Full-sized GroE complex consisting of two rings formed by seven GroEL monomers each (grey and red) and one ring of seven GroES monomers (green). (**b**) GroEL 14-mer. (**c**) GroEL heptamer ring. Shown is its end-on view. One of the monomers is shown in blue. (**d**) GroEL monomer separately, its apical domain is shown in beige. (**e**) GroEL apical domain separately. The published structure of *T. thermophilus* GroEL available in pdb-bank (PDB 2C7D) was used. The program I-TASSER™ Software (“PROGRAM”) (https://zhanggroup.org/I-TASSER/, accessed on 18 February 2022) [13,14,15] was used for manipulations with the structure.

GroEL monomer is subdivided into three domains: the apical, intermediate, and equatorial, with distinct functions in each [7]. The apical domain is responsible for the binding of GroES and of substrate proteins. The intermediate domain acts as a hinge between the apical and equatorial ones during cooperative conformational changes in the quaternary structure that accompany GroE functioning. The equatorial domain contains the ATP-binding site [8,9]; it also provides most of the lateral interactions defining formation of a heptamer as well as those between the two rings [7]. There is an engineered form of GroEL with four mutations (R452E, E461A, S463A, and V464A) in amino acid residues that participate in inter-ring interactions [10], which prevents the formation of 14-mer complex. GroEL then exists in a single-ring form, termed SRl, which was used in the studies of GroE complex functioning [11,12]. Figure 1 illustrates the forms of GroEL that find practical applications.

## 2. Direct Use of Unmodified Chaperones

It is a very attractive idea to use natural properties of chaperones in biotechnology processes. Many works aiming to exploit these properties deal with chaperones “as they are”; that is to say, co-expressing them with proteins of interest to increase the yield or with proteins prone to aggregation to support them in a soluble stable state. The enhancement of soluble expression in the presence of co-expressed chaperones was described for sarcosine oxidase from *Thermomicrobium roseum* [16], cyclodextrin glycosyltransferase (CGTase) of *Bacillus macerans* [17], humanized single-chain antibody [18], and other proteins in *E. coli*. In yeast, there is an example of expression of functional xylose isomerase in *Saccharomyces cerevisiae* [19]. In [20], coexpression of the GroE complex assisted folding of simultaneously over-expressed maltodextrin lucosidase and yeast mitochondrial aconitase, both of which are prone to aggregation. The approach proved useful in many instances; so over time, a number of plasmid systems have been developed to allow co-expression of various combinations of chaperones together with the target proteins [21,22,23,24,25,26]. There is an example of a study of the GroE contribution to inclusion bodies processing *in vivo* [27], also aiming to increase the *in vivo* recovery of a prone to aggregation protein; in this case, recombinant human tumor necrosis factor-related apoptosis-inducing ligand.

## 3. Engineered GroEL Forms

### 3.1. GroE Complex

Direct fusions of target proteins to chaperones are described in [28]. The authors expressed mouse prion protein fused in frame to the N-termini of *E. coli* chaperones DnaK or GroEL, and obtained it in large amounts in a soluble form, while normally it is insoluble in bacteria, and obtained similar results with a fragment of insoluble *Varicella Zoster* virus protein ORF21p. The authors explained this effect by an increase in the HSP local concentration within the proximity of the folding target protein, and later published a work describing the use of a set of plasmid vectors that allow the expression of recombinant proteins as cleavable N-terminal fusions with DnaK (Hsp70) and GroEL (Hsp60) of *E. coli* [29]. Another example is the expression of *Shigella*’s IpaB antigen, usually insoluble, as an N-terminal fusion with *S. typhi* GroEL, in order to produce a recombinant vaccine candidate molecule against *Shigella* infection [30]. Still, the examples of such fusions are not very numerous, which is probably due to steric difficulties arising with the addition of a full-sized protein at the N-terminus of each GroEL monomer.

The quaternary structure of GroEL forms the cavity that bears inside it the substrate-binding surface meant for the interaction with unstable hydrophobic polypeptides. It is a temptation to construct a fusion in such a way that a target unstable polypeptide would be able to interact with the substrate-binding surface. However, that is impossible to achieve by fusing target inserts to the N- or C-termini of GroEL, which leads to think about inserts into the appropriate position of the GroEL polypeptide chain. There was a work where groEL gene was subjected to insertion mutagenesis using transposon ISlacZ/in [31]. Obtained insertion mutants did not retain the ability to fold properly, and three out of four were totally or partially degraded. It was concluded that GroEL with polypeptide sequences inserted into its polypeptide chain cannot act as a chaperone, but these results may also be explained by the random manner of mutagenesis instead of a structure-oriented one. In the work of Furutani et al. [32], a GroEL type II, *Thermococcus* sp. KS-1 chaperonin alpha-subunit, with rings formed by eight monomers each, was used to create a construct of four monomers fused head-to-tail and GFP at the C-terminus of the construct. The obtained tetramers formed double-ring structures with green fluorescence. The same approach was used to express inside the cavity two prone to aggregate antibody fragments. In such setting two target proteins attached to each ring were expressed inside the cavity formed by eight TCP monomers. Here arise considerations about the limitations put by the size of the cavity—it is clear that if a relatively large target protein is fused to each monomer, the particle will not be able to assemble. For *E. coli* GroEL, by far the most studied, the size of the cavity was assessed and shown to be sufficient to accommodate a large protein. One of the reports shows binding of 116 kDa beta galactosidase with *E. coli* GroEL [33].

**Figure 2:**
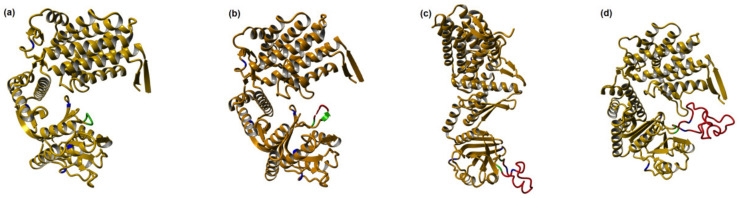
Consecutive changes in *T. thermophilus* GroEL as a carrier for peptide biosynthesis: (**a**) Initial *T. thermophilus* GroEL monomer. Methionine residues are shown in blue, amino acid residues 199–201 for introducing the polylinker are shown in green. (**b**) The introduced polylinker (corresponding amino acid residues Gly-Ser-Lys-Leu-Glu-Phe) is shown in red. (**c**) Modified *T. thermophilus* GroEL with polyphemusin I incorporated into its polypeptide chain. (**d**) Modified *T. thermophilus* GroEL with enfuvirtide incorporated into its polypeptide chain. To illustrate the position of inserts, the published structure of *T. thermophilus* GroEL available in pdb-bank (PDB 1SRV) was used. The program I-TASSER™ Software (“PROGRAM”) (https://zhanggroup.org/I-TASSER/, accessed on 18 February 2022) [13,14,15] was used for manipulations with the structure.

We have developed an approach for polypeptide biosynthesis using GroEL monomer as a carrier. In these fusion constructs, the target peptide is incorporated into the GroEL polypeptide chain in such a way that in case of formation of the correct structure, the peptide would protrude into the cavity and interact with the substrate-binding surface without hampering the oligomer structure. The idea was to obtain many advantages: peptides are difficult to express individually in cells and fusion strategy is often employed to obtain them efficiently; the chaperone would maintain hydrophobic or labile ones; the size of peptides allows them to be fused to each GroEL monomer and still not destroy the quaternary structure; being secluded inside the cavity, the peptides would not interact with the cell environment, which might allow the biosynthesis of toxic peptides, for instance, of peptide antibiotics. Steps of such a construct’s design are shown on Figure 2. Figure 2 illustrates the introduced changes using the published structure of *T. thermophilus* GroEL as a base, and I-TASSER to model the introduction of peptides into a GroEL.

As a starting point, we chose GroEL from thermophilic organism *Thermus thermophilus* in hope that it would retain its original thermostability after all subsequent changes, as, in fact, it did. In this thermophilic GroEL, we substituted all the methionine residues for leucine ones. This was performed in order to facilitate further purification of the CNBr cleaved target peptide [34]. Then, the small unstructured loop in the apical domain between amino acid residues 199–201 was chosen to introduce the insert so that it could not derange the structure and would be situated near the substrate-binding surface. On Figure 2a, methionine residues of *T. thermophilus* GroEL are shown in blue, and the loop between amino acid residues 199–201, in green. Between the codons encoding amino acids 199 and 201, we introduced the polylinker consisting of BamHI, HindIII, and EcoRI restriction sites, which added six amino acid residues Gly-Ser-Lys-Leu-Glu-Phe to the polypeptide. They are shown in red on Figure 2b. This construct was ready for cloning of target peptides flanked by methionine residues, for subsequent CNBr cleavage.

We will describe here two constructs with target peptides whose features contrast in every aspect. Polyphemusin I [35], one of the most potent antibacterial peptides, is a short (18 amino acid residues) positively charged structured peptide represented by an amphipathic beta-hairpin connected by a type IV beta-turn [36] (Figure 2c). Opposite to it, enfuvirtide, a 36 amino acid long therapeutic peptide used in treatment of AIDS patients, bears a slight negative charge and is unstructured (Figure 2d) (Enfuvirtide biosynthesis in thermostable chaperone-based fusion. Zenin V., Yurkova M., Tsedilin A. and Fedorov A., submitted in *Biotechnology Reports*). In both cases, GroEL with incorporated peptide was expressed with high yield in a soluble state. In both cases, the constructs retained GroEL’s initial thermostability and could be partially purified from host proteins by heating cell lysates; the identity of target peptides after CNBr cleavage was asserted by mass-spectrometry. On the other hand, there was a difference: the construct containing polyphemusin I was expressed in the form of an assembled 14-mer complex, thus shielding the cell environment from peptide’s toxicity and allowing its production, while the construct containing enfuvirtide was expressed mainly in the form of monomers. The explanation lays primarily in the difference in peptides’ net charges: polyphemusin I has pI 10.33, while enfuvirtide has pI 4.30. GroEL’s own pI is 5.16, i.e., it bears the same charge as enfuvirdtide, and the opposite to polyphemusin I. As a result, we observe the stabilization of the tetradecameric complex by inclusion of polyphemusin I (and also, in fact, of its 39 amino acid long dimer, unpublished data). For the toxic peptide, the maintenance of the integrity of GroE complex is vital, it could not be expressed if exposed to the cell environment [36]; however, probably, it is not so very necessary in the cases of non-toxic, but labile or hydrophobic peptides. The short conclusion about using this expression system is—of course, the exposure of the target peptide to the cell environment may be undesirable, but the net result is individual and depends on the nature of the target.

### 3.2. Monomers

HSP60, the GroEL counterpart of *Micobacterium* sp., is one of the few exceptions that exist naturally not as a tetradecameric structure, but rather as a dimer, which was shown both by native gel electrophoresis and protein cross-linking for *M. tuberculosis* chaperonin 60.1 [37] and by the crystal structure for *M. tuberculosis* chaperonin 60.2 [38,39]. This feature allows using it as a fusion partner for different polypeptides in need of stabilization without reference to the quaternary structure. Another feature of mycobacterial HSP60 is its immunogenicity. Chaperones as a part of Bacillus Calmette–Guérin (BCG) vaccine, consisting of live attenuated *Mycobacterium bovis*, are ones of its most potent immune-stimulating components. Mycobacterial chaperones are able to stimulate monocytes [37], which makes it possible to use them as vaccine adjuvants for immune boosting, and such attempts are being made. For example, one of the most common causes of cervical cancer and anal dysplasia is human papillomavirus type 16 (HPV16) [40]. There is a preventive vaccine “Gardasil”, containing L1 viral capside protein, which is already available and can prevent infection, but does not treat it [41]. Still, the data of the Centers for Disease Control and Prevention for 2018 report that there are about 43 million HPV infected at risk of malignant transformation. There is a need for therapeutic vaccine against E6 and E7 viral carcinogenic transformation proteins [42]. DNA vaccination in that case may be insufficiently immunogenic because of the viral mechanisms for evading host recognition. As a result, there are several strategies that use fusion constructs with other antigens to enhance immunogenicity. Two studies of fusions of E7 protein of HPV16 and heat shock protein 70 [43] or 65 [44] have been conducted recently; the latter one has reached the second phase of clinical trials.

### 3.3. Minichaperone

The limited use of full-sized chaperones as fusion partners, or carriers in fusion systems, can be explained by steric complications that arise with the addition of polypeptide sequences to those of chaperones: it is clear that if chaperone’s structure is deranged, then the chaperone cannot function as such. In this direction, successful use of modified minichaperone (GrAD, GroEL Apical Domain) as a fusion partner was reported [45]. The results can be explained by the use of minichaperone, first described in the laboratory of Fersht [46], as a framework for a carrier in fusions. The minichaperone has a stable structure [47] and retains GroEL’s substrate-binding surface [46,48]; it exists as a monomer, so there is no quaternary structure critical for its functionality. As for activity, the minichaperone was shown to facilitate the refolding of rhodanese and cyclophilin A in the absence of ATP and to catalyze the unfolding of native barnase [46]. In the same laboratory, the minichaperone was engineered, and the paper published describing the mutations that stabilize its structure [49], and another paper described the salt bridges stabilizing the structure of the minichaperone’s analog from the thermophilic eubacterium *T. thermophilus* [50].

**Figure 3:**
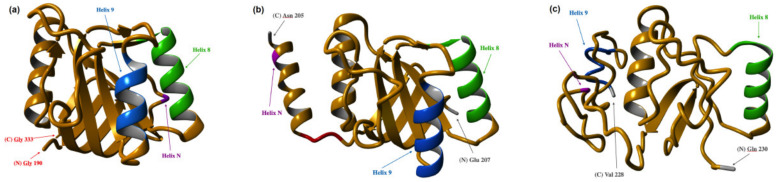
GrAD (**a**) and its permutated variants. The substrate-binding surface is formed by the helices 8, 9, and N (shown in green, blue, and purple, respectively). In (**b**,**c**), the three amino acid long linker connecting initial N- and C-termini is red. For manipulations with the structure (PDB 1SRV) the program I-TASSER™ Software (“PROGRAM”) (https://zhanggroup.org/I-TASSER/, accessed on 18 February 2022) [13,14,15] was used.

GrAD, designed as a carrier for fusion systems, is a modified minichaperone (aa residues 190 to 333). It differs from the original one (aa residues 191 to 345, [46]) by its source—it is an apical domain of *T. thermophilus* GroEL and retains its thermostability—and by replacement of all the methionine residues by leucine ones. In the fusions with GrAD, the use of a long flexible linker between the two parts of the fusion allowed the targets to find optimal position relative to the substrate-binding surface of GrAD. Targets were not model peptides, but two initially insoluble proteins, both candidates for potential development of corresponding vaccines, namely, E6 from human papilloma virus type 16, and the N-terminal fragment of E2 from hepatitis C virus. Both fusions were expressed insoluble, but could be renatured with the yield over 90%, after which they not only remained stable in high concentrations in native buffers, but could withstand freezing and lyophilization. That work had a sequel [51] aimed to improve—or rather personalize—the interactions between the target and the carrier. The work consisted of creating GrAD’s permutations, connecting its natural N and C-termini with a short linker, and making new termini at different parts of GrAD’s surface, which is illustrated on Figure 3 using the published structure of *T. thermophilus* GroEL apical domain and I-TASSER to model the newly created termini.

GrAD’s natural N- and C-termini are situated far from its substrate-binding surface formed by the helices 8, 9, and N (Figure 3a), and even with the use of a long flexible linker it may be not always possible to achieve the optimal mutual orientation of the two parts of a fusion due to arising steric impediments. The positions of new N- and C-termini for both permutations were chosen in close proximity to GrAD’s substrate-binding surface, but with a view as not to disturb the structure. Thus, for the permutation shown in Figure 3b, the new termini were placed in the unstructured flexible loop after the helix N of the original polypeptide chain; Glu 207 became the N-terminus, and Asn 205 the C-terminus. For the permutation shown on Figure 3c, the new termini were placed between the helices 8 and 9, which form major surface recognizing substrate polypeptides [50]. In this case, Glu 230 became the N-terminus, and Val 228 the C-terminus. Such a strategy allows the targets fused through a long flexible linker to the new termini to find the optimal position for the interaction with the substrate-binding surface, thus assuring their maximal stability and solubility. The selection of the optimal fusion remains, of course, individual in every case of target protein.

## 4. Refolding *In Vitro*

The above-mentioned GrAD fusions with initially insoluble proteins [45,51] were also expressed as insoluble, and effectively over 90% renatured later *in vitro*. In this case, GrAD acted as a tool for *in vitro* refolding “in cis”, since neither E6 from human papilloma virus type 16 nor N-terminal fragment of E2 from hepatitis C virus would renature without assistance. Another way is to use chaperones for refolding *in vitro* “in trans”, meaning bound to a solid matrix to act in oxidative refolding chromatography. In this case, minichaperone, GroEL’s isolated apical domain, is also often used as one of the folding devices. The binding of chaperones to a matrix may be carried out in different ways. For example, in [52], minichaperone and two foldases (DsbA and humanpeptidyl-prolyl *cis-trans* isomerase) were immobilized on a cation exchanger through poly-arginine C-terminal tail and assisted in the refolding of denatured and reduced RNase A and cyclohexanone monooxygenase, both of which contain many cysteine and proline residues, in a batch. Both proteins were recovered in soluble form with full enzyme activity with a high yield of 73% and 53%, respectively. In [53], minichaperone and oxidoreductases DsbA and DsbC were fused to a carbohydrate-binding module and immobilized on microcrystalline cellulose particles in equimolar amounts. A column with such a matrix significantly improved the oxidative chromatographic refolding of lysozyme. Moreover, chaperones immobilized on cellulose retained their functionality in denaturing conditions.

## 5. GroEL as a Scaffold

Chaperonin’s ability to support and stabilize other proteins finds yet another very direct application in studying the structures of proteins and peptides while they are bound to GroEL. This approach was found suitable for NMR, X-rays, and cryo-electron microscopy techniques [54,55,56,57,58]. The work [59] describes the advances in the application of GroEL biosensor biolayer interferometry (BLI) technologies and includes expanded uses of GroEL as a molecular scaffold for electron microscopy determination.

Another side of GroEL activity attributed to its apical domain is its ability to suppress the formation of fibrils. It was shown in [60] that Gly192, at the hinge II site that connects the apical domain with the intermediate domain in GroEL, plays a pivotal role in the dynamic apical domain movement, where, later on, the mutation of Gly192 to a tryptophan results, besides other effects described in [60], in a loss of activity of the chaperonin toward fibrillogenic peptides because its apical domain is disoriented [61]. Then, this residue was substituted with amino acid residues of varying van der Waals volumes with the intent to modulate the affinity of GroEL toward fibrillogenic peptides [62], and it was shown that while GroEL affinity increased in accordance to the larger van der Waals volume of the substituent amino acid side chain, the effects of the chaperonin on α-synuclein fibrillation were different: the wild-type chaperonin caused changes in both the initial lag phase and the rate of fibril extension, whereas the effects of the G192*X* mutants were more specific toward the lag phase.

In addition, GroEL itself shows the ability to form nanofibers. In the works of Chen et al., it was shown that despite a pronounced inhibitory effect on the fibril growth, both GroEL and its isolated apical domain demonstrate the propensity to form amyloid-like fibrils increasing under acidic conditions [63]. Later on, the group found that in the presence of sodium dodecyl sulfate at a submicellar concentration, GroEL oligomers turn into modular structural units, which are observed to self-assemble into cylindrical nanofibers in a physiological buffer. In addition, through targeted mutagenesis where two cysteine residues were introduced at the entry site of the GroEL cage, the authors found that the formation of GroEL nanoassembly could be modulated depending on the redox condition of incubation. Such tunable GroEL nanofibers, in the opinion of the authors, may have broad applications [64].

## 6. GroEL as an Adjuvant and Antigen

Aside from their crucial involvement in protein folding, chaperones may also be immunogenic [65,66], as has already been mentioned about mycobacterial HSP60. Chaperones, and particularly GroEL counterparts of some pathogenic bacteria, can relocate to the bacterial cell surface, and mediate adhesion to mucin during the initial interaction steps before initiating colonization and invasion, or they could be secreted [67,68]. In this way, a chaperone becomes detectible for the immune system and thus can be used as an antigen. HSPs interact with the innate immune system via Toll-like and scavenger receptors [67]. Then they can promote phagocytosis and maturation of dendritic cells, which enhances processing and the presentation of an antigen inducing adaptive immunity [68]. These considerations serve as the basis for using chaperones in vaccine technology.

One of serious medical problems is currently antimicrobial resistance due to microbes quickly evolving protective mechanisms against antibiotic drugs [69]. Chaperones have strongly conserved sequences that differ among bacterial species [70]. This feature could be effectively used in subunit vaccines designed against bacteria causing diseases that are difficult to cure. A subunit vaccine consists of one or more highly immunogenic surface antigens of a pathogen, and thus can be developed based on a pathogen’s chaperone. For example, there are studies of recombinant *Helicobacter pylori* GroEL and an immunogenic epitope isolated from it as a candidate vaccine against *H. pylori*, a bacterium causing chronic gastritis and most peptic ulcer diseases for which there is no specific treatment. Such a candidate vaccine showed induction of the adaptive antibody-mediated immunity in mice [71,72].

## 7. Beyond GroEL

The choice of chaperones for biotechnological applications is not, of course, limited by GroEL and its different forms, and in this review, dedicated to GroEL, there has already been mentioned a joint use of several chaperones for co-expression *in vivo* and for refolding chromatography *in vitro*. Now, it would be appropriate to outline the involvement of other chaperones in the development of vaccines and treatments.

DnaK is a conservative and multi-epitope chaperone like GroEL, and can also be used to generate immune response against bacteria. Currently available vaccines against typhoid fever caused by *Salmonella* serotype *typhi* cannot induce cellular immunity along with neutralizing antibodies, while in [73] it was shown that DnaK induced T-cell response together with IgA, IgM, and IgG required for protection against *Salmonella* infection in mice. IgA is the prime antibody subtype of mucosa defenses, which is broadly present in the gastrointestinal and respiratory tracts, the main gateways of infection; the titer of specific IgG was observed even after 10 months after the last immunization. Thus, in this case, DnaK proved a promising vaccine candidate.

Chaperones can be used for animal vaccines, too, based on the same principles. Bacterial fish infections are a serious problem for fish farming, and chaperone-based vaccines are being developed against *Edwardsiella tarda* [74], *Vibrio harveyi* [75], and a bacteria of the genus *Nocardia* causing nocardiosis [76].

Therapeutic vaccines, unlike preventive ones, are rather immunotherapeutic preparations intended to stimulate the immune system of a patient with already existing illness in order to cure it. The need of therapeutic vaccines arises in case of chronic diseases or oncology. Hepatitis C virus infection is a good example. HCV-infected people often are initially asymptomatic or have non-specific symptoms, and in many cases the disease turns into a chronic form. Quite a low percent of antibodies are neutralizing, because HCV antigens can effectively hide from immunity, and the development of a therapeutic vaccine is a challenge. In [77], it was reported that a fusion construct consisting of Hsp27-NS3 from *Mus musculus* and HR9 and the Cady-2 complex for protein penetration could significantly stimulate effective cell-mediated immunity via conserved sequence at positions 1251–1259 of NS3 and lead to viral clearance in mice. Moreover, there are studies investigating the possibility of using chaperones in cancer therapy [78,79]. Certain tumors can express different antigenic fingerprints. This feature was used in Allovax^®^, an anti-cancer patient-specific vaccine that is based on chaperone-rich cell lysate of the patient’s tumor. It has reached the II phase of clinical trials (Immunovative Therapies, Ltd. Jerusalem, Israel. ITL-020-HENK-VAXPII) [80].

Despite promising approaches of chaperone applications in vaccines and treatment, there are some pitfalls ahead. In some cases, isolated chaperones can be contaminated with LPS and LPS-associated molecules from bacteria [68]. Often this is the reason for non-reproducible results. So, it is crucial to confirm the apyrogenicity of tested substances. In addition, the induction of immunity by HSPs purified from tumors can be dependent on the maintenance of HSP-bound peptide [68], and the therapeutic effect is associated with a high enough concentration of this peptide, which is difficult to control. Earlier this problem was the main reason for the rejection of Vitespen approval [68]. Moreover, subunit vaccines with bacterial chaperones may impact the commensal microbiome of the host species due to antibody cross reactivity, and in some cases, there is a risk of autoimmune events [81]. Hence subunit vaccines should be evaluated for cross reactivity and its potential implications [67].

Neurodegenerative diseases are also in the spotlight of chaperone-based treatment. It is a natural approach, because the Alzheimer’s disease, Parkinson’s disease, and tauopathies are mediated by misfolded proteins [82]. There are many studies on the treatment of these diseases with chaperones by host-enhanced synthesis or the introduction of recombinant ones with varying degrees of success [83,84,85,86].

The attempts at enhancing chaperones activity to combat detrimental protein misfolding and aggregation involve two approaches: directed evolution and rational design [87]. Directed evolution consists in creating many chaperone variants by random mutagenesis followed by selection of one or more variants with better substrate recognition; in this case, substrates being misfolded proteins that cause neuronal degeneration. It turned out that only two missense mutations of GroEL, and one of GroES, led to the changes in specific substrate binding [88]. For the studied chaperones, among them Spy [89,90], GroEL/S [88], DnaK [91], Hsp104 [92], the increase in specificity was accompanied by their increased flexibility and reduced stability. There are also attempts to expand the hydrophobic surface area by substituting a charged polar amino acid residue for a hydrophobic one [90]. Some of the corresponding Hsp104 variants with disaggregase activity also had increased ATPase activity [88,93]. Moreover, surprisingly, some of them had a toxic effect on yeast [93]. Another direction in engineering of chaperones is a rational design, for example, with the use of grafted amyloid-motif antibodies (gammabodies) [94]. Fibril assembly inhibition properties of Hsp70 were enhanced by adding to its C-terminus a sequence of complementary peptide from selected gammabodies epitope [95]. Still, to find more effective directions for chaperone’s engineering, physical principles, and mechanisms of chaperone-induced disaggregation of amyloids, tau-fibrils, or protein aggregates should be elucidated.

## 8. Conclusions

Even a short review demonstrates almost unlimited possibilities of applying chaperones for versatile and sometimes very ingenious uses. On the other hand, practical uses are deterred where there is not enough information about fundamental laws underlying such a wide range of chaperones’ properties, from folding to unraveling of protein aggregates. Chaperones themselves are intricately organized proteins, and their engineering has met, of course, many unpublished drawbacks, in addition to published successes. It seems that practical developments would benefit from fundamental studies, which would open wide fields for further research.
